# Assessment of Spatial Agglomeration of Agricultural Drought Disaster in China from 1978 to 2016

**DOI:** 10.1038/s41598-019-51042-x

**Published:** 2019-10-07

**Authors:** Qian Wang, Yang-yang Liu, Yan-zhen Zhang, Lin-jing Tong, Xiaoyu Li, Jian-long Li, Zhengguo Sun

**Affiliations:** 1 Department of Ecology, School of Life Sciences, Nanjing University, Nanjing, 210023 China; 2 College of Agro-Grassland Sciences, Nanjing Agricultural University, Nanjing, 210095 China

**Keywords:** Sustainability, Natural hazards

## Abstract

Drought disaster space agglomeration assessment is one of the important components of meteorological disaster prevention and mitigation. Agriculture affected by drought disaster is not only a serious threat to world food security, but also an obstacle to sustainable development. Additionally, China is an important agricultural import and export country in the world. Therefore, we used the global Moran’s I and the local indicators of spatial autocorrelation (LISA) to reveal the spatial agglomeration of agricultural drought disaster in China from1978 to 2016, respectively. The results showed that China’s agricultural drought disaster presents local spatial autocorrelation of geographical agglomeration at national level during the study period. The spatial agglomeration regions of China’s agricultural drought disaster were in Inner Mongolia, Jilin province, Heilongjiang province, Liaoning province, Shanxi province, Hebei province, Shandong province, Shaanxi province and Henan province, indicating that agricultural drought disaster mainly distributed in North and Northwest China, especially occurred in the Yellow River Basin and its north areas. We also found that the overall movement direction of agricultural drought disaster agglomeration regions was northwest, and the maximum moving distance was 722.16 km. Our results might provide insight in early warning and prevention for drought disaster.

## Introduction

Drought as one of the worldwide natural phenomena can occur not only in arid and semiarid regions, but also in wet areas^1,2^. At present, there is no unanimous definition of drought. Conventional scientific literature recognizes four types of drought: meteorological, hydrological, agricultural, and socioeconomic^3–5^. More than one half of the earth is susceptible to drought each year^6,7^. Because drought can impact many sectors of the society and environment^8^, which is a limiting factors affecting human survival and social stability. Therefore, drought has attracted much attention from academia and governments all over the world.

Great progress has been made in drought field, numerous specialized indexes have been developed and widely used to monitor and quantify evaluate the different types of drought. According to the previous studies, the Palmer Drought Severity Index (PDSI) (Palmer, 1965) is the earliest drought index in the world. The Standardized Precipitation Index (SPI) has been recommended as a key meteorological drought index by the World Meteorological Organization^9–11^. In addition, other drought indexes are also widely used in drought studies through compare the applicability of drought indexes. Besides, some drought hazard models are used to assess the drought in different places of the world. Scholars have studied many aspects of drought used above methods, such as duration^12,13^, mechanism^14^, classification^15,16^, severity^13,17,18^, spatial extent^19,20^, the overall change trend and periodic oscillation of the drought^21^.Undeniable, those studies made process in drought studies. Because the drought has the relationship with precipitation, climate, and other factors, as one of the natural phenomena, drought has spatial and temporal dimensions. Recently, there has been an increasing interest in the space-time of drought. However, there has been little discussion about the geographical agglomeration of the drought disaster, especially about the geographical agglomeration of agricultural drought disaster.

Agricultural drought disaster is one of the four types of the drought^3,4,22^, which may affect the food production or food quality, and even ultimately impact on the food security and social stability. It is well known that food security is the fundamental to human survival and social stability. Thus, we focused on the agricultural drought disaster in our study.

China is a significant agricultural country of the world and has become the fifth largest agricultural exporter and the fourth largest agricultural importer in the world since 2001^23^. As an agricultural country with typical monsoon climate, the severe drought in China has caused tremendous losses during the last decade^24^. Therefore, agricultural drought disaster was the stress factor affecting the sustainable development of China’s agriculture and environment. In recent years, there were many researchers studied the local agricultural drought disaster in China. For example, a study used the Integrated Surface Drought Index (ISDI) for agricultural drought disaster monitoring in mid-eastern China^25^. Furthermore, others used the index or model to assess agricultural drought disaster^26–28^. Besides, assessment of agricultural drought vulnerability also conducted research in North of China^29^. However, most studies in agricultural drought disaster have carried out in partial area of China. There little study in agricultural drought disaster at national level, especially about the geographical agglomeration of agricultural drought disaster. Moreover, China’s agriculture situation may impact the sustainable development of the agriculture and environment in China even and affect the food security of the world. In other words, for sustainable development, we must reduce the potential impact of drought disaster on agriculture. It is well known that reliable drought disaster early warnings are important for developing plans to reduce the potential impact of drought disaster on agriculture. Thus, discovering the geographical agglomeration of agricultural drought disaster plays an important part in drought disaster monitoring and early warning. Besides, historical drought studies can provide a very valuable basis for explaining the current drought disaster behavior^30^. To discovering the geographical agglomeration of China’s agricultural drought disaster, we collected statistical data of agricultural drought disaster 1978 to 2016.

The Moran’s I statistic could be said to be the most widely used method of test area spatial autocorrelation^31^. According to the previous studies, the Moran’s I was widely used in phase separating mixtures^32^, analysis on urban traffic status^33^,and pollution hotspot analysis, *et al*. To date, there was barely study in drought disaster using the Moran’s I. Furthermore, the concept of “center of gravity” comes from physics, its draws on analogy with Newton’s Law of Gravitation^34^. Since the gravity can reflect the dynamic changes in spatial distribution, at present, gravity has long been one of the most successful models in economics^35^, immigration^36,37^, highway^38^, environmental pollution monitoring^39^, and other fields^40^. However, there was few studies used the center of gravity migration model into drought disaster. To discover the geographical agglomeration and dynamic changes of drought disaster plays an important part in drought monitoring and early warning. Therefore, we used the Moran’s I and the center of gravity model to study agricultural drought disaster, which might provide a perspective for the study of drought disaster.

In this study, the main goal was to mapping the geographical agglomeration of China’s agricultural drought disaster based on the historical drought disaster data from 1978 to 2016 at the national scale. Moran’s I involving global and the local Moran’s I^41^, is often used to measure the data clustering level^42^. Here we analyzed China’s agricultural drought disaster using the global Moran’s I and the local Moran’s I at national level, respectively. In addition, we also calculated the center of gravity of the high cluster regions based on the center of gravity migration model. The results may help the government and farmers to take some scientific measures at early stages of agricultural drought disaster to reduce the potential impact on agriculture^43,44^.

## Materials and Methods

### Study area description

China is an agricultural country that is easily affected by natural hazards^45^. There are four types natural disasters occur in China, which is drought disaster, flood disaster, wind disaster and low temperature freezing disaster. According to the statistics data of agricultural disaster areas from 1978 to 2016, the average annual areas covered of drought disaster, flood disaster, wind disaster and low temperature freezing disaster was approximately 2.27 × 10^7^ hm^2^, 1.09 × 10^7^ hm^2^, 4.48 × 10^6^ hm^2^, 3.19 × 10^6^ hm^2^, respectively^46^. Thus, drought disaster accounts for approximately 53% of the total, that is, greater than half of the agriculture was affected by drought disaster. Additionally, China’s agriculture drought disaster still increases in the future with the climate changing^30,47^. In other words, China faces a high risk of drought disaster. Based on the statistics data collected by the Ministry of Water Resources of the People’s Republic of China (http://www.mwr.gov.cn/), total of 26 provinces (regions) suffered drought disaster in 2017. In addition, the total agricultural areas affected by drought disaster was 1.82 × 10^7^ hm^2^, of which areas covered of agricultural drought disaster was 9.95 × 10^6^ hm^2^, areas affected of agricultural drought disaster was 4.5 × 10^6^ hm^2^, and no harvest areas was 7.5 × 10^5^ hm^2^. Because of the impact of the drought disaster, the food loss, and the economic crop loss was approximately 1.34 × 10^10^ kg, 116.84 billion Yuan, respectively.

### Data

The agricultural drought data were collected from three aspects that were areas covered of agricultural drought disaster (hm^2^), areas affected of agricultural drought disaster (hm^2^), and grain loss (kg). To evaluate the drought disaster impact on China’s agriculture, we obtained the data from the Ministry of Water Resources of the People’s Republic of China (http://www.mwr.gov.cn/). Those agricultural data were reliable and widely used in agriculture research in China.

### Methods

#### Spatial autocorrelation model

Spatial autocorrelation denotes the conditions where more similar attributes are found among neighboring observations than those far away^48^.That is, spatial autocorrelation analysis reveals the distribution characteristics and internal relationships of spatial objects. The global Moran’s I and the local Moran’s I are the most commonly used test statistic for spatial autocorrelation with map patterns^49^.

The global Moran’s I is the typical method of spatial autocorrelation with value from −1 to 1. If I = 0, means a random spatial pattern with no spatial autocorrelation. Furthermore, positive values suggest spatially clustered patterns in adjacent sites, while negative values indicate that samples reveal very different values from the neighboring ones. Additionally, to identify localized clustering, the local indicators of spatial autocorrelation (LISA) were also calculated. Four categories of LISA were as follows: high-high cluster, high-lower outlier, lower-high outlier and lower-lower cluster. High-high cluster means high drought disaster values are surrounded by high values; high-lower outliner suggests that a high drought disaster value surrounded by low values; and lower-high outlier indicates that a low drought disaster value surrounded by high values; moreover, lower-lower cluster denotes low drought disaster values are surrounded by low values.

#### Center of gravity migration model

Geographic objects can represent their spatial dynamics through the center of gravity trajectory. The center of gravity coordinates are usually expressed in longitude and latitude, which calculated as follows^50^:

1 $$\overline{X}=\frac{\mathop{\sum }\limits_{i=1}^{n}{P}_{i}{X}_{i}}{\mathop{\sum }\limits_{i=1}^{n}{P}_{i}}$$

2 $$\overline{Y}=\frac{\mathop{\sum }\limits_{i=1}^{n}{P}_{i}{Y}_{i}}{\mathop{\sum }\limits_{i=1}^{n}{P}_{i}}$$

where $\overline{X}$ and $\overline{Y}$ are the longitude and latitude of the center of gravity of drought disaster region *i*, respectively; *n* is the number of drought disaster regions; and *X*_*i*_ and *Y*_*i*_ are the latitude and longitude centroids of drought disaster region *i*, respectively; and *P*_*i*_ is drought disaster region *i*.

The space distance of the center of gravity calculated by follows:

3 $${D}_{i-j}={\rm{C}}\ast \sqrt{{({X}_{j}-{X}_{i})}^{2}+{({Y}_{j}-{Y}_{i})}^{2}}$$

*D*_*i–j*_ is the distance between drought disaster region *i* and drought disaster region *j*; and C is a constant of 111.111 km.

## Results

### The overall the spatial characteristics of china’s agricultural drought disaster

To evaluate the overall spatial characteristics of China’s agricultural drought disaster, we calculated the average global Moran’s I. The average global Moran’s I of China’s agricultural drought disaster was represented in different periods, respectively (Table 1). A positive global Moran’s I value indicates that the tendency of the spatial characteristics of China’s agricultural drought disaster towards clustering. However, a negative global Moran’s I value indicates that the tendency of the spatial characteristics of China’s agricultural drought disaster towards dispersion. As shown in Table 1, the average global Moran’s I value of areas covered of agricultural drought disaster was positive, suggesting that the tendency of the spatial characteristics areas covered of China’s agricultural drought disaster toward clustering. Similarly, the average global Moran’s I value of areas affected of agricultural drought disaster was greater than zero, indicating that areas affected of China’s agricultural drought disaster also has geographical agglomeration. In other words, the overall spatial characteristics of China’s agricultural drought disaster were non-random but present a spatial autocorrelation of geographical agglomeration during the study period.

**Table 1:** The average global Moran’s I of China’s agricultural drought disaster in different periods.

	Areas covered of drought disaster	Areas affected of drought disaster
1978–1984	0.280	0.248
1986–1989	0.336	0.355
1991–1997	0.323	0.322
1999–2004	0.445	0.426
2006–2010	0.398	0.438
2013–2016	0.362	0.341

### The spatial agglomeration of china’s agricultural drought disaster at national scale

To assess the spatial agglomeration of agricultural drought disaster in China, we calculated the average of the local indicators of spatial autocorrelation (LISA). The calculated results can be divided into four different spatial patterns: high-high cluster, high-lower outlier, lower-high outlier and lower-lower cluster.

**Figure 1:**
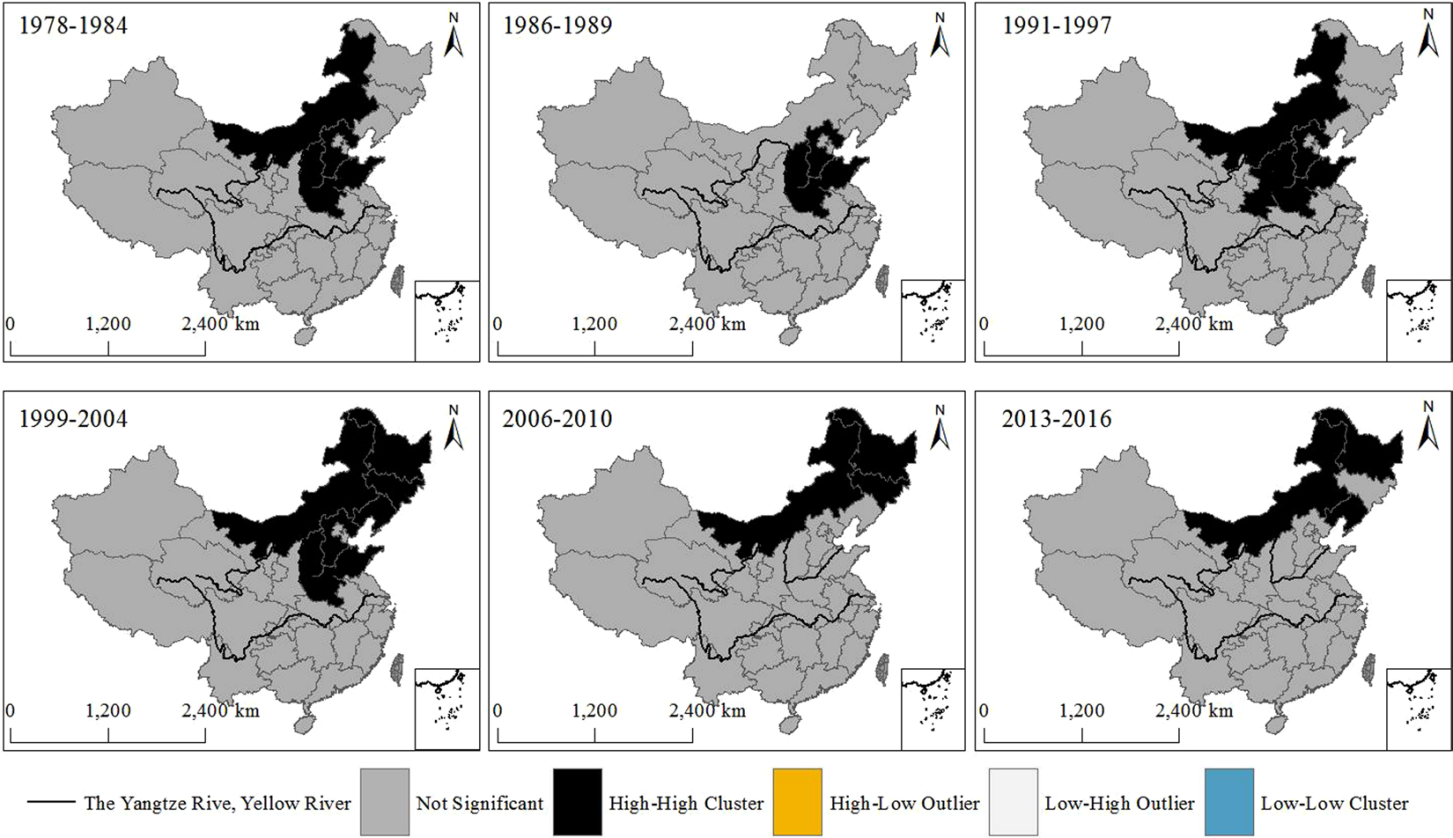
The spatial agglomeration of areas covered of China’s agricultural drought disaster from 1978 to 2016. This figure was produced using ArcGIS 10.2.

From Fig. 1, the results showed that there was only high- high cluster (high drought occurrence rate) spatial pattern of areas covered of agricultural drought disaster in each period. During the period 1978–1984, the high-high clusters were mainly located in Shanxi province, Hebei province, Shandong province, Henan province and Inner Mongolia. During the period 1986–1989, the high-high clusters were chiefly distributed in Hebei province, Shandong province, Shanxi province and Henan province. The high-high clusters were mainly in Inner Mongolia, Shanxi province, Hebei province, Shandong province, Shaanxi province and Henan province during the period 1991–1997. In addition, the high-high clusters were primarily located in Inner Mongolia, Jilin province, Heilongjiang province, Liaoning province, Shanxi province, Hebei province, Shandong province and Henan province during the 1999–2004. Similarly, the high-high clusters were chiefly occured in Inner Mongolia, Jilin province and Heilongjiang province during the 2006–2010. The high-high clusters were primarily distributed in Inner Mongolia, Heilongjiang province and Liaoning province during the 2013–2016. Thus, the spatial agglomeration of areas covered of agricultural drought disaster has spatial differences and presents local spatial autocorrelation of geographical agglomeration in China during the study period.

**Figure 2:**
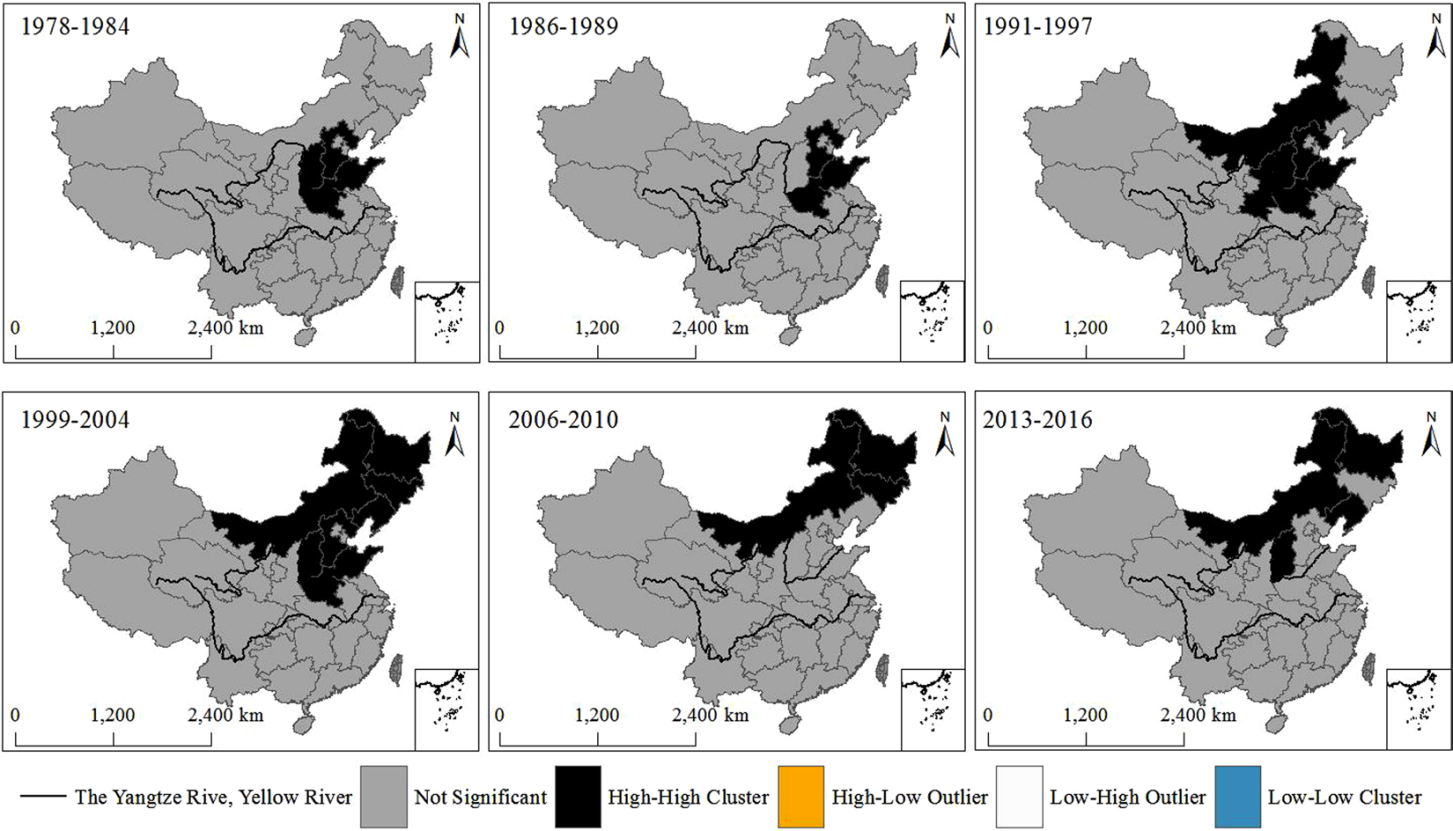
The spatial agglomeration of areas affected of China’s agricultural drought disaster area from 1978 to 2016. This figure was produced using ArcGIS 10.2.

Besides, to find where with either high or low values cluster spatially, we analyzed the spatial-temporal evolution of areas affected of agricultural drought disaster area in China from 1978 to 2016. From Fig. 2, the results showed that there was only high- high cluster (high drought occurrence rate) spatial pattern of areas affected of agricultural drought disaster area in each period. During the period 1978–1984, the high-high clusters were primarily distributed in Shanxi province, Hebei province, Shandong province and Henan province. During the period 1986–1989, the high-high clusters were mainly located in Hebei province, Shandong province and Henan province. The high-high clusters were primarily distributed in Inner Mongolia, Shanxi province, Hebei province, Shandong province, Shaanxi province and Henan province during the period 1991–1997. In addition, the high-high clusters were mainly in Inner Mongolia, Jilin province, Heilongjiang province, Liaoning province, Shanxi province, Hebei province, Shandong province and Henan province during the 1999–2004. Similarly, the high-high clusters were chiefly occurred in Inner Mongolia, Jilin province and Heilongjiang province during the 2006–2010. The high-high clusters were primarily concentrated in Inner Mongolia, Heilongjiang province, Liaoning province and Shanxi province during the 2013–2016. Therefore, the spatial agglomeration of areas affected of agricultural drought disaster area has spatial differences and presents local spatial autocorrelation of geographical agglomeration in China.

### The center of gravity of agricultural drought disaster agglomeration regions and its spatial movement

The location and trajectory of the geographic object’s center of gravity is an important indicator that reflects the dynamic changes in spatial distribution^51^. We calculated the center of gravity of China’s agricultural drought disaster agglomeration regions to find their dynamic changes in spatial distributions. The north azimuth, and the distance of the center of gravity was calculated, respectively.

**Figure 3:**
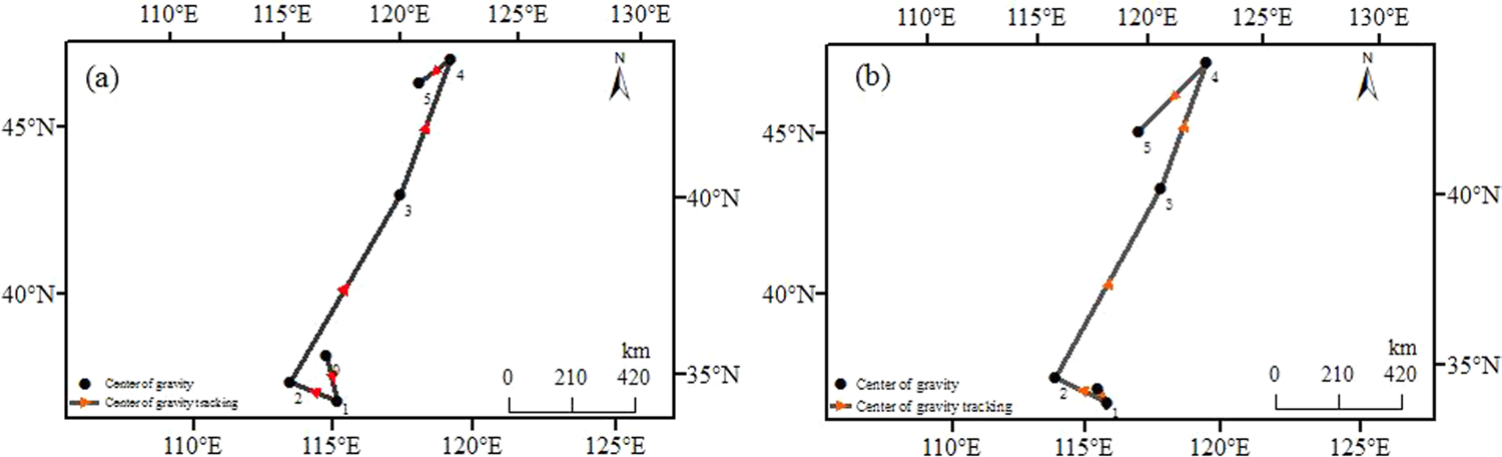
The mean center tracking of agricultural drought disaster agglomeration regions from 1978 to 2016 **(a)** the areas covered of agricultural drought disaster **(b)** areas affected of agricultural drought disaster drought. This figure was produced using ArcGIS 10.2. Note: 0 stands for the center of gravity during the period 1978–1984; 1 stands for the center of gravity during the period 1986–1989; 2 stands for the center of gravity during the period 1991–1997; 3 stands for the center of gravity during the period 1999–2004; 4 stands for the center of gravity during the period 2006–2010; 5 stands for the center of gravity during the period 2013–2016. The same below.

The direction of movement of the center of gravity of drought disaster agglomeration regions was shown as follows: southeast-northwest-northeast-northeast-southwest (Fig. 3). Furthermore, the center of gravity was mainly concentrated in the northeast China, especially in Inner Mongolia. The center of gravity in Inner Mongolia region accounts for approximately 60% of the total. Moreover, the north azimuth, and the distance of the movement was shown in Table 2. The maximum distance of movement of China’s agricultural drought disaster agglomeration regions was 722.16 km, and the minimums distance of movement was 57.87 km.

**Table 2:** The north azimuth, and the distance of the movement of agricultural drought disaster agglomeration regions from 1978 to 2016.

	Areas covered of drought disaster		Areas affected of agricultural drought	
	North Azimuth (Degree)	Distance(km)	North Azimuth(Degree)	Distance(km)
1	167.26	152.96	146.07	57.87
2	−68.45	165.29	−64.59	192.42
3	30.47	699.13	29.26	722.16
4	20.74	463.70	19.62	448.48
5	233.20	129.02	224.08	321.82

## Discussion

### Methodology

Why did we choose the Moran’s I to study drought disaster? A possible explanation for this might be that the Moran’s I statistic is arguably the most widely applied method for testing spatial autocorrelation in areal datasets^52^. In addition, drought disaster has the characteristics of the spatial autocorrelation. Besides, the Moran’s I have been widely used to study the spatial structure of data in many fields except for drought disaster. Another possible explanation for this is that compare to the drought index, the results of the Moran’s I are visualized, we can easy to find the disaster agglomeration areas. Therefore, we used the Moran’s I to study the drought disaster in our study.

In this study, we used the local Moran’s I to analysis China’s agricultural drought disaster at the national scale from 1978 to 2016. Our results showed that the high-high clusters of the agricultural drought disaster were mainly distributed in Inner Mongolia, Jilin province, Heilongjiang province, Liaoning province, Shanxi province, Hebei province, Shandong province and Henan province (Figs 1, 2). According to the agricultural acreage and food production, the Chinese government has designated 13 provinces (regions) as the main agricultural provinces since 2004, which are Hebei province, Inner Mongolia, Liaoning province, Jilin province, Heilongjiang province, Jiangsu province, Anhui province, Jiangxi province, Shandong province, Henan province, Hubei province, Hunan province and Sichuan province. Our results showed that the provinces (regions) affected by the drought disaster were consistent with the agricultural areas. Those results supported that the spatial distribution of agricultural drought disaster analyzed by Moran’s I was reasonable. Therefore, the Moran’s I could be used to study the drought disaster.

### Analysis the spatial agglomeration of china’s agricultural drought disaster

Since drought has spatial and temporal dimensions, finding areas where drought accumulates may help people take measures in advance to reduce the losses affected by the drought. Our results found that the high-high clusters of the provinces (regions) affected by the drought disaster in our study mainly occurred in the Yellow River Basin and its north areas. Our findings were consistent with the agricultural areas,which also agreed with the previous studies reported^53^.

There are several possible explanations for this result. The differences in natural climatic factors is the main factor leading to the loss of agricultural drought disaster^54,55^. Due to the large east-west span in China, the regional climate is significantly different. Therefore, the regional climate is inevitably a factor influencing the spatial distribution of drought disaster. In addition, the monsoon climate due to thermal differences between land and sea is particularly significant in China. The previous study reported that the south wind had experienced a strong period from 1958 to 1976 and then a weak period from 1977 to present^56^. As a result, the precipitation distribution over northern China during the concerned periods altered obviously due to changes of the water vapor supply^56,57^. Moreover, the temperature has increasing trend in northern China since the 1990’s^58^. Since precipitation and temperature are the most direct and important hazard factors for crops, the reduction of crop production is largely the result of these two factors^59^. Because summer drought occurs in the critical period of crop growth, crop production is greatly affected. Thus, the northern China’s agricultural drought disaster has a relationship with the summer south wind.

### Analysis the spatial movement of agricultural drought disaster agglomeration regions

The center of gravity of the high concentration regions reflected the dynamic changes of agricultural drought disaster. Our results showed that the overall movement direction of agricultural drought disaster agglomeration regions was northwest, and the maximum moving distance was 722.16 km. A possible explanation for this might be that regional differences in precipitation and temperature during different periods were responsible for the movement of the center of gravity. Another possible explanation for this was that reasonable irrigation was the cause of the shift in the focus of agricultural drought disaster. Besides, changes in the ecological environment might also cause the shift in the focus of agricultural drought disaster. We also found that the center of gravity in Inner Mongolia region accounts for approximately 60% of the total. This result may be explained by the fact that drought disaster in Inner Mongolia, and Northeast China had increased significantly over the past 30 years. Especially in the early 21st century, there was a severe drought with a long duration and widely influence that is very rare for half a century of history^60^. That is to say, the degree of agricultural drought disaster was the main factor causing the shift of the center of gravity.

## Conclusions

We used the Moran’s I to reveal the spatial agglomeration pattern of the China’s agricultural drought disaster. Some of the important conclusions derived are given below:

We analyzed the spatial-temporal evolution of China’s agricultural drought disaster based on the Moran’s I at the national scale. Our result showed that China’s agricultural drought disaster presents local spatial autocorrelation of geographical agglomeration during the study period. The high-high clusters were mainly in Inner Mongolia, Jilin province, Heilongjiang province, Liaoning province, Shanxi province, Hebei province, Shandong province, Shaanxi province and Henan province during the study period. The regional climate was the factor influencing the spatial distribution of drought disaster.

We also found that the overall movement direction of the agglomeration center of gravity was northwest, and the maximum moving distance was 722.16 km. The degree of agricultural drought disaster was the main factor causing the shift of the center of gravity.

Based on the results, the Moran’s I can be a useful method to analyze the spatial autocorrelation of agricultural drought disaster. In other words, we can use the Moran’s I to assess the spatial autocorrelation of other natural disasters in our future study. However, China has a complex climate and different natural disasters. We only analyzed the drought disaster affected on the China’s agriculture. In our future work, we will use the model to analysis other natural disasters affected on the China’s agriculture.
